# Active Surveillance Cultures and Procalcitonin in Combination With Clinical Data to Guide Empirical Antimicrobial Therapy in Hospitalized Medical Patients With Sepsis

**DOI:** 10.3389/fmicb.2022.797932

**Published:** 2022-04-07

**Authors:** Silvia Spoto, John Daniel Markley, Emanuele Valeriani, Antonio Abbate, Josepmaria Argemi, Roshanak Markley, Marta Fogolari, Luciana Locorriere, Giuseppina Beretta Anguissola, Giulia Battifoglia, Sebastiano Costantino, Massimo Ciccozzi, César Bustos Guillén, Silvia Angeletti

**Affiliations:** ^1^Diagnostic and Therapeutic Medicine Department, Campus Bio Medico University of Rome, Rome, Italy; ^2^Division of Infectious Disease and Epidemiology, Department of Internal Medicine, Virginia Commonwealth University, Richmond, VA, United States; ^3^Central Virginia, Veterans Administration Hospital, Richmond, VA, United States; ^4^Division of Cardiology, Department of Internal Medicine, Pauley Heart Center, Virginia Commonwealth University, Richmond, VA, United States; ^5^Liver Unit Clinica Universidad de Navarra Hepatology Program, Center for Applied Medical Research (CIMA), IdiSNA, Universidad de Navarra, Pamplona, Spain; ^6^Unit of Clinical Laboratory Science, Campus Bio Medico University of Rome, Rome, Italy; ^7^Unit of Medical Statistics and Molecular Epidemiology, Campus Bio Medico University of Rome, Rome, Italy; ^8^Division of Infectious Diseases, Department of Internal Medicine, Clinica Universidad de los Andes, Santiago, Chile

**Keywords:** MDRO colonization, antimicrobial resistance (AMR), nasal and rectal surveillance swab, sepsis, procalcitonin, systemic inflammatory response syndrome (SIRS), quick Sequential Organ Failure Assessment (qSOFA), Sequential Organ Failure Assessment (SOFA)

## Abstract

**Objective:**

The prevalence of colonization with multidrug-resistant organisms (MDRO) has increased over the last decade, reaching levels as high as 23% in certain patient populations. Active surveillance cultures (ASC) represent a valuable tool to identify patients colonized with MDRO to apply preventive measures, reduce transmission, and guide empiric antimicrobial therapy. There is a paucity of data evaluating the impact of admission ASCs to predict future infection. The aim of this study was to evaluate the concordance between ASCs results and the development of clinical infection by the same microorganism identified in the surveillance swab (“swab-related infection”), in hospitalized septic patients, and to evaluate the presence of specific risk factors associated with the development of a swab-related infection.

**Methods:**

All adults admitted to the Diagnostic and Therapeutic Medicine Department of the University Hospital Campus Bio-Medico of Rome with a diagnosis of infection or any other medical reason with admission surveillance swabs (rectal or nasal) between January 2018 and February 2021 were included in the study. A retrospective chart review was conducted to identify patients that developed infections with concordant MDROs identified on ASC, and the risk factors for swab-related infection. Secondary outcomes were need of intensive care unit transfer, length of stay, sepsis or septic shock development, and all-cause mortality.

**Results:**

A total of 528 patients were included in the study, of which 97 (18.3%) had a positive surveillance swab. Among patients with positive surveillance swabs, 18 (18.5%) developed an infection with the same microorganism recovered from the swab, 57 (58.8%) developed an infection with a different microorganism than that recovered from the surveillance swab, and 22 (22.7%) did not develop an infection during hospitalization. The number of colonized sites, an interventional procedure within the previous 3 months, a Systemic Inflammatory Response Syndrome (SIRS) score ≥ 2, and a quick Sequential Organ Failure Assessment (q-SOFA) score ≥ 2 were associated with a significantly higher risk of developing a swab-related infection. SIRS and q-SOFA scores ≥ 2 and procalcitonin ≥ 0.43 ng/ml help for identifying patients with a swab-related infection.

**Conclusion:**

Patients with positive surveillance swabs were at increased risk for development of infections by the same MDRO identified in surveillance swabs (swab-related infection). This study is the first to show that the positivity of surveillance swabs, in combination with anamnestic data, PCT values, and SIRS or q-SOFA scores, serves as a valuable tool to help clinicians predict patients at higher risk for swab-related infection development and guide the administration of appropriate empiric antimicrobial therapy in septic patients.

## Introduction

Antimicrobial resistance refers to the capability of some bacteria or fungi to resist to specific antimicrobial therapies (CDC, 2019).

Resistant pathogens including *Clostridioides difficile* and ESKAPE bacteria (*Enterococcus faecium*, *S. aureus*, *K. pneumoniae*, *Acinetobacter baumannii*, *Pseudomonas aeruginosa*, and *Enterobacter* spp.) are responsible for more than 2.8 million infections in the United States, a doubled length of stay, and increased mortality (35–70% of infected patients) (Lübbert et al., 2014; Pisney et al., 2014; Tischendorf et al., 2016; Cella et al., 2017; Angeletti et al., 2018; Cancilleri et al., 2018; De Florio et al., 2018; CDC, 2019).

The proportion of patients with unfavorable outcomes and death increase with the spectrum of antimicrobial resistance (AR) ranging from 3.3% for methicillin-resistant *Staphylococcus aureus* (MRSA), to 5.7% for extended spectrum beta-lactamase (ESBL)-producing *Enterobacteriaceae*, ∼8% for carbapenem-resistant *Enterobacteriaceae* (CRE), or *Acinetobacter* or multidrug-resistant *Pseudomonas aeruginosa*, and up to 9.9% in vancomycin-resistant *Enterococcu*s (VRE) (CDC, 2019).

The prevalence of colonization with multidrug-resistant organisms (MDRO) progressively increased over the last 10 years, reaching 16.5% in 2016 and up to 23% in high-risk populations [oncohematologic patients or hospitalized in an intensive care unit (ICU)] (Lübbert et al., 2014; Pisney et al., 2014; Martin et al., 2016; Tischendorf et al., 2016). In our hospital, the estimated incidence of healthcare-associated infection (HAI) secondary to MDRO was 2.4 per 1,000 in-hospital days with more than 10% of these infections occurring in patients on internal medicine wards.

Prolonged ICU stay, need for mechanical ventilation, prior antimicrobial use, severe chronic comorbidities, and immunodeficiency are well-known risk factors for development of infection from colonization (Borer et al., 2012; Papadimitriou-Olivgeris et al., 2013). Infection development, however, mainly occurring in the lung, urinary tract, skin and soft tissue, and bloodstream, depends on the complex interaction between pathogen and host. Bloodstream infection (BSI) is facilitated by bacterial translocation from colonized sites or by contiguous contamination from skin (Wiest and Garcia-Tsao, 2005; Tischendorf et al., 2016; Shimasaki et al., 2019).

Identifying the presence of colonization by MDRO and applying preventive measures may reduce the occurrence of unfavorable outcomes and is associated with a 28% reduction in overall mortality (CDC, 2019). Previous studies showed that surveillance cultures may predict subsequent infections by MDRO (Papadomichelakis et al., 2008). In one study in the ICU setting, the colonization concordance rates between clinical and surveillance cultures were 70, 82, and 88% in the setting of Gram-negative intra-abdominal infection, ventilator-associated pneumonia (VAP), and BSI, respectively (Blot et al., 2005; Reddy et al., 2007; Papadomichelakis et al., 2008; Baba et al., 2011; MacFadden et al., 2018). A more intensive active surveillance culture (ASC) strategy (twice-weekly tracheal aspirates in intubated patients or pharyngeal swabs in non-intubated patients and once-weekly rectal swabs) increased the predictive power (Papadomichelakis et al., 2008). Furthermore, the shorter the time between positive surveillance cultures and infection, the better the concordance (Hayon et al., 2002).

In this context, the use of ASC represents a meaningful tool to predict development of infection and to improve antimicrobial stewardship. However, performance of ASC is cost and labor intensive and should be reserved for patients at high risk for colonization with MDRO, including residents of long-term-care facilities, those with neutropenia, those hospitalized within the previous month or receiving antibiotics within the previous 3 months, or those with age > 70 years (Siegel et al., 2017; Tseng et al., 2017). The Surviving Sepsis Guidelines advise incorporating prior culture data and known colonization into decisions for empiric antibiotic therapy, although data are limited and weak and adherence to this recommendation is unknown (Evans et al., 2021). Based on the high prevalence of MRSA infection in the United States (more than 50% of *S. aureus* isolates), the Infectious Diseases Society of America (IDSA) recommends empiric anti-MRSA therapy for hospital-acquired or ventilator-associated pneumonia (Mandell et al., 2007; Kalil et al., 2016; Weiner et al., 2016; Rhodes et al., 2017; Parente et al., 2018; Mergenhagen et al., 2020; Evans et al., 2021).

Recent data, furthermore, suggest that negative results in nasal swabs may guide antibiotics de-escalation for pneumonia (ATS/IDSA, 2019).

Conversely, data on resistant pathogen-related intra-abdominal, urinary tract, and wound infection are limited (Solomkin et al., 2010; Acquisto et al., 2018). IDSA guidelines indicate that MRSA coverage may be indicated in infected patients who have had multiple abdominal surgeries (Solomkin et al., 2010).

Biomarkers of infection such as neutrophil-to-lymphocyte and platelet-to-lymphocyte ratios (NLR), procalcitonin (PCT) value, and mid-regional pro-adrenomedullin (MR-proADM) value may help in diagnostic management, stratification of disease severity, and prognosis (Angeletti et al., 2016a,b; Spoto et al., 2018, 2019, 2020a,b, 2021). The highest diagnostic and prognostic ability was observed at the following cutoff values: NLR ≥ 7.97, PCT ≥ 0.5 ng/ml, and MR-proADM ≥ 1.50 nmol/l, as previously reported (Angeletti et al., 2016a,b; Spoto et al., 2018, 2019, 2020a,c, 2021). The diagnostic ability of these biomarkers significantly increased when the SIRS, q-SOFA, and SOFA scores were added to the model (Angeletti et al., 2016a,b; Spoto et al., 2018, 2019, 2020c, 2021).

The aim of this study was to evaluate the concordance between ASC results and the development of clinical infection by the same microorganism identified in the surveillance swab (“swab-related infection”), in hospitalized septic patients, and to evaluate the presence of specific risk factors associated with the development of a swab-related infection.

## Materials and Methods

This retrospective study was conducted between January 2018 and February 2021 and was approved by the Ethical Committee of the University Hospital Campus Bio-Medico of Rome (28.16TS Com Et CBM). Informed consent was not required for the retrospective design of the study.

### Study Design and Patients’ Selection and Outcomes

The study was conducted at a 380 acute-care bed University Hospital in Italy (Campus Bio-Medico of Rome).

Patients included in the study were all patients admitted to the Diagnostic and Therapeutic Medicine Department of the University Hospital with a diagnosis of infection or any other medical reason, in whom nasal and rectal surveillance swabs were obtained because they had a high risk of colonization by resistant bacteria, accordingly with local infection control policy (Tseng et al., 2017). These patients were retrospectively and consecutively recruited.

The patients were defined as “at high risk of colonization by resistant bacteria,” when they were admitted from the emergency, medical, or surgical departments or the ICU or have been hospitalized within the previous month, accordingly with the local infection control policy (Tseng et al., 2017), based on the national multidrug-resistant pathogen epidemiology.

Local infection control policy indicates that ASCs (rectal and nasal) should be obtained from all patients considered at high risk of colonization by resistant bacteria including those admitted from the emergency, medical, or surgical departments or the ICU or have been hospitalized within the previous month (Tseng et al., 2017). Contact and/or precaution measurements were used for patients with positive results of nasal or rectal surveillance cultures. Clinical cultures were obtained when an infection is suspected. PCT and MR-proADM were measured at hospital admission and during hospitalization, as required, in all patients with a clinical suspicion of infection or to exclude infection.

We included for analysis all patients with a positive result of nasal or rectal surveillance swab and excluded patients < 18 years old, pregnant women, and those with no surveillance swab results.

Baseline patient characteristics were retrospectively collected from medical records including demographic information (age, sex category, BMI), swab-related information (data of sample, site of colonization, microbiological results), presence of comorbidities (cardiovascular, pulmonary, kidney, liver disease), immune status (active malignancy or other causes of an immunosuppression), treatments (corticosteroids, chemotherapy, radiotherapy, antibiotics), interventional procedures or hospitalizations within the previous 3 months, laboratory values (complete blood count, PCR, PCT, MR-proADM), clinical scores (e.g., SIRS, q-SOFA, SOFA), presence and type of infection, and microbiological results.

A clinical evaluation was performed in all patients at admission and at least three times a day during hospitalization by an Internist specialized in infectious disease who followed a methodological algorithm for the diagnosis and treatment of infection as previously reported (Spoto et al., 2020a). This algorithm includes the identification of the source of infection, aggressive source control, and the administration of tailored antimicrobial therapy incorporating patient-related and etiologic risk factors and PCT values (Spoto et al., 2019). A “Hit early and hit hard” antimicrobial therapy strategy was used in colonized patients presenting with critical illness and hemodynamic instability according to local antibiogram data, source of infection, PCT values, and antibiotic exposure or hospitalization within the previous month (Mehta et al., 2014; Tamma et al., 2020; Spoto et al., 2020a).

The primary outcome was identifying swab-related infections, and risk factors for swab-related infections in patients with positive ASCs. Secondary outcomes were need of ICU transfer, length of stay, sepsis or septic shock development, and all-cause mortality.

### Definitions, Laboratory, and Microbiological Parameters

“Colonization” refers to patients carrying MDRO (*K. pneumoniae*, *P. aeruginosa*, *A. baumannii*, *S. aureus*, *E. faecalis/E. faecium*) without signs or symptoms of an infection but with the capability to spread microbes to others (Martin et al., 2016).

“Swab-related infection” refers to an infection developed by the same MDRO identified in the surveillance swab. Diagnosis of sepsis and septic shock was performed according to the Third Consensus Conference Criteria of 2016 when a q-SOFA or SOFA scores were ≥ 2 from baseline in the presence of an infection. Diagnosis and treatment of pneumonia and urinary tract, intra-abdominal, skin, soft tissue, bloodstream, and all other included infections were managed according to the available international guidelines (ATS, 2005; Mandell et al., 2007; Solomkin et al., 2010; Dennis et al., 2014; CDC, 2016; Singer et al., 2016; Siegel et al., 2017).

Complete blood count (CBC) was performed on whole blood by *Sysmex* XE-9000 (Dasit, Cornaredo, Italy) following the manufacturer’s instruction. NLR was calculated by the ratio between absolute values of neutrophils and lymphocytes. CRP was measured by Alinity c (Abbott Diagnostics, Abbott Park, IL, United States) following the manufacturer’s instruction. PCT and MR-proADM plasma concentrations were measured by an automated Kryptor analyzer, using a time-resolved amplified cryptate emission (TRACE) technology assay (Kryptor PCT; Brahms AG, Hennigsdorf, Germany) with commercially available immunoluminometric assays (Brahms) (Angeletti et al., 2016a,b, 2018; Cella et al., 2017; Cancilleri et al., 2018; De Florio et al., 2018; Spoto et al., 2018, 2019, 2020c, 2021).

Blood specimens from patients were collected in BACTEC bottles containing anaerobic or aerobic broth and resins. Blood culture bottles were incubated in a BACTEC FX instrument (Becton Dickinson, Meylan, France) until they resulted positive for bacterial growth or for a maximum of 5 days. Positive samples were cultivated in selective agar media. Growing colonies were identified by MALDI-TOF Brahms) (Angeletti et al., 2016a,b, 2018; Cella et al., 2017; Cancilleri et al., 2018; De Florio et al., 2018; Spoto et al., 2018, 2019, 2020a, 2021). Selective and nonselective media were used for microbiological cultures.

Nasal and rectal swabs were performed with BBL™ Culture Swab™. After collection, samples were inoculated directly to MacConkey agar and a chromogenic agar plate for CRE, VRE, and MRSA screening. All isolated bacteria were identified by a MALDI-TOF mass spectrometer (Bruker Daltonics, Billerica, MA, United States). The antimicrobial susceptibility was performed by Phoenix Instruments (BD, Beckton Dickinson, Franklin Lakes, NJ, United States), and MICs were interpreted according to Clinical and Laboratory Standards Institute (CLSI) M100-ED30 breakpoints.

### Statistical Analysis

Categorical variables were expressed as counts and percentages and compared using the chi-squared or Fisher’s exact tests, as appropriate. Continuous variables were expressed as mean (standard deviation) or median (interquartile range) according to data distribution after applying the Wilk–Shapiro test and compared using the Student’s *t*-test or the Mann–Whitney *U*-test, as appropriate.

Patients with positive results of surveillance swab were classified by the presence of a swab-related infection (Group 1), the presence of an infection by a pathogen different from those detected by swab (Group 2), or the absence of an infection during hospitalization (Group 3).

In the primary analysis, a stepwise logistic regression model was performed including clinically relevant variables as regressor (age, sex category, presence of comorbidity, number of colonized sites, prior antibiotics use, prior interventional procedures, clinical risk scores, and inflammatory biomarkers).

Receiver operating characteristic (ROC) analysis was performed among independent variables associated with infection to define the cutoff point for SIRS, q-SOFA, SOFA score, and PCT values.

Data were analyzed using MedCalc 11.6.1.0 statistical package (MedCalc Software, Mariakerke, Belgium) and R (version 3.6.3, R Core Development Team, Vienna, Austria)^1^.

## Results

We obtained ACS of 528 patients admitted to the Diagnostic and Therapeutic Medicine Department from the emergency department (71.2%), other medical departments (8.2%), ICU (6.2%), or surgical department (3.1%), or from those who had been hospitalized within the previous month (11.3%), from a period since January 2018 to February 2021.

Patients were classified in three different groups: Group 1 included all patients with a swab-related infection (patients in whom the clinical infection was caused by the same MDRO identified as colonizer in the swab); Group 2 included all patients that developed an infection by a different pathogen from that detected in the swab; and Group 3 included all patients colonized by MDRO who did not developed any infection during hospitalization.

**FIGURE 1:**
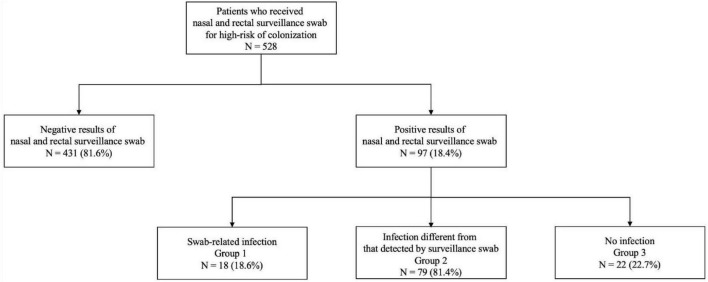
Flowchart of the study groups.

Overall, 431/528 (81.6%) had a negative surveillance swab and did not develop any infection. Conversely, 97/528 (18.4%) had a positive surveillance swab. Of these, 18/97 (18.6%) patients belonged to Group 1, while the remaining 57/97 (58.7%) patients corresponded to Group 2 and 22/97 (22.7%) patients belonged to Group 3 (Figure 1).

In Groups 1 and 2, the median global frametime for developing an infection was 8 days: 6 days for Group 1 and 11 days for Group 2.

### Baseline Characteristics of the Included Population

Table 1 shows the baseline patient characteristics distributed by Groups 1–3. Across all groups, the median age was similar (80.5 vs. 81.0 vs. 78.5 years) and nearly half were male (50.0 vs. 56.1 vs. 68.2%). Nearly two-thirds of patients in all groups were admitted from the emergency department (72.2 vs. 68.4 vs. 77.3%) while the remaining were admitted from a medical department (11.1 vs. 10.5 vs. 0%), surgical department (5.6 vs. 1.8 vs. 4.5%), or the ICU (11.1 vs. 3.5 vs. 9.1%) or had been hospitalized within the previous month (0 vs. 15.8 vs. 9.1%).

**TABLE 1:** Demographic characteristics, clinical scores, and inflammatory biomarkers of the study population classified into three different groups: Group 1, Group 2, and Group 3.

Variables	Group 1	Group 2	Group 3	*p*

	**Surveillance swab positive**	**Surveillance swab positive**	**Surveillance swab positive**	

	**Presence of swab-related infection**	**Presence of other infection than swab**	**Absence of infection**	

	***N* = 18**	***N* = 57**	***N* = 22**	
Age, median [IQR]	80.50 [75.25, 83.00]	81.00 [72.00, 87.00]	78.50 [70.25, 81.00]	0.252
Sex category, male (%)	9 (50.0)	32 (56.1)	15 (68.2)	0.476
**Anamnestic variables, *n* (%)**				
Cancer	5 (27.8)	16 (28.1)	5 (22.7)	0.886
COPD	5 (27.8)	25 (43.9)	11 (50.0)	0.342
Cardiovascular disease	14 (77.8)	46 (80.7)	20 (90.9)	0.477
Liver disease	2 (11.1)	7 (12.3)	2 (9.1)	0.922
Chronic kidney disease	13 (72.2)	29 (50.9)	8 (36.4)	0.077
Diabetes mellitus	3 (16.7)	18 (31.6)	9 (40.9)	0.253
Cerebrovascular disease	11 (61.1)	27 (47.4)	6 (27.3)	0.091
Autoimmune disease	7 (38.9)	6 (10.5)	3 (13.6)	0.017
Autoimmune therapy	6 (33.3)	6 (10.5)	2 (9.1)	0.040
Previous antibiotic therapy	12 (66.7)	28 (49.1)	5 (22.7)	0.017
Previous hospitalization	12 (66.7)	28 (49.1)	8 (36.4)	0.162
Previous interventional procedure	7 (38.9)	10 (17.5)	1 (4.5)	0.020
Current interventional procedure	5 (27.8)	21 (37.5)	10 (45.5)	0.517
Number of colonized sites > 1	5 (27.8)	3 (5.3)	0 (0.0)	0.003
BMI > 25	2 (11.1)	9 (15.8)	6 (27.3)	0.354
BMI < 25	11 (61.1)	16 (28.1)	7 (31.8)	0.035
SIRS ≥ 2	16 (88.9)	14 (24.6)	7 (31.8)	< 0.001
SOFA ≥ 2	15 (83.3)	40 (70.2)	14 (63.6)	0.381
q-SOFA ≥ 2	7 (38.9)	2 (3.5)	2 (9.1)	0.001
PCT, median [IQR]	1.11 [0.44, 3.54]	0.17 [0.08, 0.51]	0.16 [0.06, 0.46]	0.011
MR-proADM, median [IQR]	2.45 [2.08, 3.62]	2.47 [1.46, 3.55]	1.93 [1.12, 2.60]	0.397
CRP, median [IQR]	11.03 [4.61, 19.15]	9.11 [2.57, 18.07]	4.87 [1.12, 13.32]	0.503
NLR, median [IQR]	7.24 [3.66, 10.95]	5.66 [3.95, 9.88]	9.29 [7.50, 11.08]	0.700
Sepsis, *n* (%)	14 (77.8)	15 (26.3)	0	< 0.001
Septic shock, *n* (%)	4 (22.2)	5 (8.8)	0	0.243
Need of ICU transfer, *n* (%)	0	1 (1.8)	0	0.746
LOS (median [IQR])	18.00 [13.25, 26.25]	11.00 [8.00, 19.00]	8.50 [7.00, 13.75]	0.005
Mortality, *n* (%)	1 (5.6)	5 (8.8)	2 (12.5)	0.775

*BMI, body mass index; COPD, chronic obstructive pulmonary disease; CRP, C-reactive protein; ICU, intensive care unit; IQR, interquartile range; LOS, length of stay; MR-proADM, mid-regional pro-adrenomedullin; NLR, neutrophil-to-lymphocyte ratio; PCT, procalcitonin; q-SOFA, quick Sequential Organ Failure Assessment; SIRS, systemic inflammatory response syndrome; SOFA, sequential organ failure assessment.*

Cardiovascular, cerebrovascular, and chronic kidney diseases were the most represented comorbidity, followed by autoimmune disease, chronic obstructive pulmonary disease, cancer, diabetes mellitus, and liver disease (Table 1).

A BMI < 25 kg/m^2^ (61.1 vs. 28.1 vs. 31.8%, *p* = 0.04), previous antibiotic treatment (66.7 vs. 49.1 vs. 22.7%, *p* = 0.02), and an interventional procedure within the previous 3 months (38.9 vs. 17.5 vs. 4.5%, *p* = 0.02) were more prevalent in Group 1, compared to the other groups.

A significantly higher proportion of patients in Group 1 had a SIRS (88.9 vs. 24.6 vs. 31.8%, *p* < 0.01) or q-SOFA (38.9 vs. 3.5 vs. 9.1%, *p* < 0.01) scores ≥ 2 and presented with sepsis (77.8 vs. 26.3 vs. 0%, *p* < 0.01) or septic shock (22.2 vs. 8.8 vs. 0%, *p* = 0.24). The proportion of patients with SOFA score ≥ 2 were similar in all three groups (83.3 vs. 70.2 vs. 63.6%, *p* = 0.38).

While the median values of PCT were significantly higher in Group 1 than in Groups 2 and 3 (1.1 vs. 0.2 vs. 0.2 ng/ml, *p* = 0.01), the median values of CRP (11.0 vs. 9.1 vs. 4.9 mg/dl, *p* = 0.50), MR-proADM (2.45 vs. 2.47 vs. 1.93 nmol/l, *p* = 0.40), and NLR (7.2 vs. 5.7 vs. 9.3) were similar across groups (Table 1).

Length of stay was higher in Group 1 than in Groups 2 and 3 (18 vs. 11 vs. 8.5 days, *p* < 0.01); only one patient in Group 2 needed an ICU admission during hospitalization, and a similar proportion of patients died in all 3 Groups (5.6 vs. 8.8 vs. 12.5 %, *p* = 0.78).

### Site of Colonization and Infection

Rectal colonization was identified in 38.9% (7/18) of patients in Group 1 (CRE *Klebsiella pneumoniae*, 3 patients; VRE, 2 patients; MDR *Klebsiella pneumoniae*, 1 patient; ESBL positive *Klebsiella pneumoniae*, 1 patient), 64.9% (37/57) of patients in Group 2 (VRE, 19 patients; ESBL positive *Klebsiella pneumoniae*, 7 patients; MDR *Klebsiella pneumoniae*, 6 patients; CRE *Klebsiella pneumoniae*, 2 patients; MDR *Acinetobacter baumannii*, 1 patients; CRE *Acinetobacter baumannii*, 1 patient; CRE *Pseudomonas aeruginosa*, 1 patient), and 59.9% of patients (13/22) in Group 3 (VRE, 6 patients; ESBL positive *Klebsiella pneumoniae*, 4 patients; MDR *Klebsiella pneumoniae*, 2 patients; CRE *Klebsiella pneumoniae*, 1 patient), while nasal colonization by MRSA was found in 22.2% (4/18), 19.3% (11/57), and 22.7% (5/22) of patients, respectively (Table 2). In Group 1 than in Groups 2 and 3, the proportion of patients with more than one site of colonization was higher (38.9%, 7/18 vs. 14.0%, 8/57 vs. 18.2%, 4/22).

**TABLE 2:** Results of surveillance swab and concordant swab-related infection.

Organism identified on surveillance swab	Total number of patients colonized	Rectal sample	Nasal sample	Total number of concordant swab-related infections
MRSA (nasal)	21	0	21	4
CRE (rectal)	8	8	0	3
VRE (rectal)	27	27	0	2
MDR (rectal)	10	11	0	1
ESBL positive (rectal)	12	12	0	1
Polymicrobial	19	19	8	7
Total	97	77	29	18/97 (18.5%)

In Group 1, 75.0% (3/4) patients with nasal MRSA colonization developed pneumonia and 25.0% (1/4) soft tissue infection. Furthermore, 66.7% (6/9) of patients with rectal colonization developed upper urinary tract infection, 11.1% (1/9) lower urinary tract infection, 11.1% (1/9) pneumonia, and 11.1% (1/9) intra-abdominal abscess.

In Groups 2 and 3 out of 10 patients (30.0%) with nasal MRSA colonization developed pneumonia, 3 (30.0%) lower urinary tract infection, 2 (20.0%) soft tissue infection, 1 (10.0%) upper urinary tract infection, and 1 (10.0%) intra-abdominal infection. Furthermore, 17 out of 44 patients (38.8%) with rectal colonization developed pneumonia, 12 (27.5%) upper urinary tract infection, 3 (6.9%) lower urinary tract infection, 3 (6.9%) intra-abdominal infection, 2 (4.5%) soft tissue infection, 1 (2.2%) spontaneous bacterial peritonitis, 1 (2.2%) septic arthritis, 1 (2.2%) catheter-related blood stream infection, 1 (2.2%) *Clostridioides difficile* infection, 1 (2.2%) surgical site infection, 1 (2.2%) endocarditis, and 1 (2.2%) phlebitis. Finally, among patients with > 1 site of colonization, 2 presented an intra-abdominal infection and 1 had endocarditis.

Overall, 7 patients (38.9%) in Group 1 and 15 patients (26.3%) in Group 2 had a BSI.

### Multivariable Regression Model and Receiver Operating Characteristic Curves

The results of a stepwise logistic regression model revealed that the presence of a positive ASC was associated with a significantly higher risk of developing an MDRO infection compared to patients with a negative ASC (OR 6.3; 95% CI, 3.5–11.2, *p* < 0.019). In the former group, however, the number of colonized sites > 1 (OR 15.1; 95% CI, 1.7–212.1, *p* = 0.02), an interventional procedure within the previous 3 months (OR 7.1; 95% CI, 1.2–60.0, *p* = 0.04), a SIRS score ≥ 2 (OR 10.8; 95% CI, 1.8–103.6, *p* = 0.02), and a q-SOFA score ≥ 2 (OR 8.4; 95% CI, 1.2–103.6; 0.04) were associated with a significantly higher risk of developing a swab-related infection (Table 3).

**TABLE 3:** Stepwise logistic regression between Group 1 vs. Group 2 and between Group 1 vs. Group 3.

Variables	Odds ratio	95% CI	*p*-value
Chronic kidney disease	3.2	0.7–19.2	0.17
Number of colonized sites	**15.1**	**1.7–212.1**	**0.02**
Immunosuppressive therapy	3.8	0.6–27.7	0.16
Previous interventional procedure	**7.1**	**1.2–60.0**	**0.04**
SIRS ≥ 2	**10.8**	**1.8–103.6**	**0.02**
q-SOFA ≥ 2	**8.4**	**1.2–103.6**	**0.04**

*CI, confidence interval; q-SOFA, quick Sequential Organ Failure Assessment; SIRS, systemic inflammatory response syndrome. Bold values correspond to statistically significant variables.*

**FIGURE 2:**
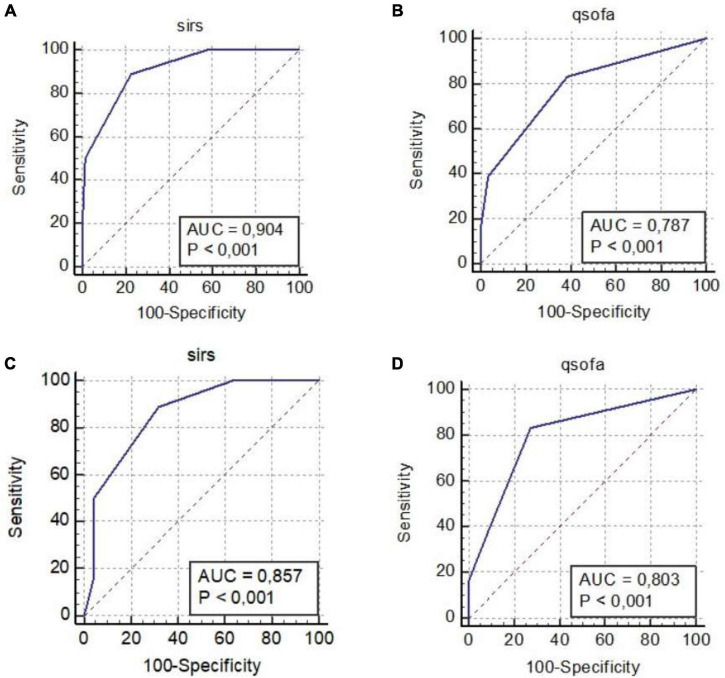
ROC curves: SIRS criteria Group 1 vs. Group 2 **(A)**, q-SOFA criteria Group 1 vs. Group 2 **(B)**, SIRS Criteria Group 1 vs. Group 3 **(C)**, and q-SOFA criteria Group 1 vs. Group 3 **(D)**. AUC, area under the curve; q-SOFA, quick Sequential Organ Failure Assessment; SIRS, systemic inflammatory response syndrome; SOFA, sequential organ failure assessment.

**FIGURE 3:**
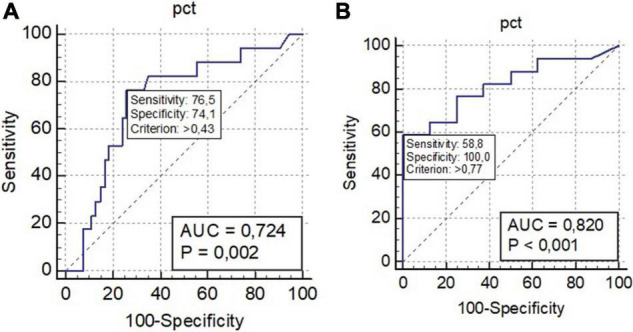
ROC curve: procalcitonin (PCT) Group 1 vs. Group 2 as unique biomarker discriminating Group 1 from Group 2 **(A)** and PCT Group 1 vs. Group 3 as unique biomarker discriminating Group 1 from Group 2 **(B)**. AUC, area under the curve; q-SOFA, quick Sequential Organ Failure Assessment; SIRS, systemic inflammatory response syndrome; SOFA, sequential organ failure assessment.

The results of the ROC curve analysis shown as SIRS or q-SOFA scores ≥ 2, and PCT ≥ 0.43 ng/ml, significantly differentiated Group 1 vs. Group 2 and Group 1 vs. Group 3 (with PCT values > 0.77; Tables 4A,B and Figures 2A–D, 3A,B).

**TABLE 4:** ROC curve analysis of SIRS, q-SOFA, SOFA scores, NLR, PCR, PCT, and MR-proADM comparing Group 1 vs. Group 2 (Panel A) and Group 1 vs. Group 3 (Panel B).

Variables	Sensitivity	Specificity	AUC	*p*-value
**Panel A: Group 1 vs. Group 2**				
SIRS ≥ 2	**50.0**	**98.3**	**0.91**	**<0.0001**
q-SOFA ≥ 2	**38.9**	**96.5**	**0.80**	**<0.0001**
SOFA ≥ 2	66.7	52.6	0.60	0.07
NLR < 12	88.9	22.8	0.50	0.95
CRP > 3.82	88.2	35.1	0.56	0.44
PCT > 0.43	**76.5**	**74.1**	**0.72**	**0.002**
MR-proADM > 2.2	75.0	48.4	0.56	0.50
**Panel B: Group 1 vs. Group 3**				
SIRS ≥ 2	**50.0**	**95.4**	**0.86**	**<0.0001**
q-SOFA ≥ 2	**39.0**	**90.0**	**0.80**	**<0.0001**
SOFA ≥ 2	66.7	45.4	0.64	0.09
NLR > 5.7	61.1	81.8	0.63	0.25
CRP > 2.45	88.2	45.5	0.63	0.26
PCT > 0.77	**58.8**	**100**	**0.82**	**< 0.001**
MR-proADM > 2.9	41.7	83.3	0.72	0.10

*AUC, area under the curve; CRP, C-reactive protein; MR-proADM mid-regional pro-adrenomedullin; NLR, neutrophil-to-lymphocyte ratio; PCT, procalcitonin; q-SOFA, quick Sequential Organ Failure Assessment; SIRS, systemic inflammatory response syndrome; SOFA, sequential organ failure assessment. Bold values correspond to statistically significant variables.*

## Discussion

Our results suggest that identification of MDRO on ASC at the time of hospital admission is associated with a significantly increased risk for developing an infection by the same microorganism during hospitalization time. This finding provides critical information to guide empiric antimicrobial management in the setting of sepsis. Colonization with MDRO among hospitalized patients has increased over the last several decades. Indeed, we identified an 18.6% rate of MDRO colonization based on ASCs in our study, which is similar to recent estimates (Tischendorf et al., 2016). Rapid identification of colonized patients represents a meaningful tool for guiding initial empirical antibiotic therapy and antimicrobial stewardship efforts.

Several factors were associated with an increased risk of swab-related infection. A positive ASC at the time of admission, the presence of SIRS or q-SOFA score ≥ 2, PCT > 0.43, number of colonized sites > 1, and the execution of an interventional procedure within the previous 3 months were independently associated with an increased risk of a swab-related infection.

Used in combination, anamnestic variables (number of colonized sites, previous interventional procedures), clinical scores, laboratory markers, and ASC results could be applied in an algorithmic way to guide the prompt administration of appropriate empiric antimicrobial therapy, resulting in decreasing mortality and length of stay (LOS) in patients with sepsis or septic shock (Spoto et al., 2020a). Implementation of this strategy among patients in our study may explain the low number of ICU transfers (1.0%) and low mortality rate (8.2%); however, additional studies are needed to determine if this approach is associated with improved outcomes (Spoto et al., 2020b).

Our study also affirms the importance of clinical scores and laboratory markers in the management of infection.

SIRS and q-SOFA scores are easily calculated and rapidly identify patients at high risk of severe infections due to sepsis and septic shock. In patients with positive ASCs or a SIRS or q-SOFA score ≥ 2, PCT > 0.43 was associated with an increased risk of swab-related infection, which aligns with prior studies linking clinical scores and PCT with severe infection.

PCT confirms its role for approaching to an infectious process with higher values in Group 1 (MDRO-infected patients) than other inflammation biomarkers such as CRP and MR-proADM that were found to be comparable in the three groups, expressing a similar state of inflammation in the 3 groups.

Indeed, in line with recent literature, the use of PCT as a marker in septic patients significantly reduces the development of infections caused by MDRO or *Clostridioides difficile*, 28-day mortality, median length of antibiotic therapy, and relative cost of hospitalization (Kyriazopoulou et al., 2021).

The results of our study confirm the suggestion of the Surviving Sepsis Guidelines that advise the implementation of prior culture data and known colonization into decisions for empiric antibiotic therapy (Evans et al., 2021).

Furthermore, this study is the first to show that the combination of the results of ASCs and PCT, in addition to clinical and anamnestic data, can guide appropriate clinical and antibiotic management. Interestingly, the SOFA score did not show a significant association with the risk of swab-related infection. This may have been secondary to the low proportion of patients in our study admitted to the ICU.

Our results are in line with previous studies in which the use of an adequate clinical management represented a key factor for a prompt diagnosis and for the administration of an appropriate empiric therapy (Dennis et al., 2014; Mehta et al., 2014; Angeletti et al., 2016a,b; CDC, 2016; Singer et al., 2016; Spoto et al., 2018, 2019, 2020a,b,c, 2021; Tamma et al., 2020).

Conversely, in our groups of patients, the duration of antimicrobial therapy, described by multiple works as a risk factor for bacterial transmigration from gut permeability, which could lead to the development of clinical infection by colonizing microorganisms, was not found (Antimicrobial Resistance Collaborators, 2022).

Inadequate antimicrobial therapy, monotherapy, and AR are extremely linked, increasing the rate of severe infections such as in the case of ESKAPE pathogens which impact on mortality and global economy (Kumar et al., 2010; Mehta et al., 2014; de Jong et al., 2016; Tamma et al., 2020; Spoto et al., 2020a).

The use of ASCs in targeted, high-risk patient populations represents a meaningful tool that may be used in daily clinical practice in order to predict swab-related MDRO infections and optimize antimicrobial therapy, and may improve other important outcomes such as cost, LOS, and mortality. Negative results of ASCs in patients without risk factors for MDRO infections facilitate the administration of standard antimicrobial regimens based on local resistance patterns. Conversely, positive ASC results facilitate the rapid administration of appropriate empiric antimicrobial therapy, as well as appropriate isolation of colonized patients, in order to minimize the risk of transmission to other patients and healthcare workers.

Our study aims at helping clinicians to recognize the patients at risk for developing sepsis or septic shock by colonizing MDRO, and to individualize an optimal empirical antibiotic therapy before blood culture or other microbiological results that could be available or give negative results for taking clinical decisions.

Between 97 patients with a surveillance swab positivity, 18/97 (18.6%) developed an infection caused by the same MDRO. Even, in this 18% of patients, a positive ASC was predictive of a clinical infection with a swab-related infection, accordingly with literature. Altogether, ASCs, anamnestic data, PCT value, SIRS, or q-SOFA clinical scores provide vital information in clinical decision making.

## Conclusion

Patients with positive surveillance swabs were at an increased risk for development of concordant MDRO infections (swab-related infection). This study is the first to show that the positivity of surveillance swabs, in combination with anamnestic data, PCT values, and SIRS or q-SOFA scores, serve as a valuable tool to help clinicians predict patients at higher risk for swab-related infection development and guide the administration of appropriate empiric antimicrobial therapy in septic patients.

## Data Availability Statement

The original contributions presented in the study are included in the article/supplementary material, further inquiries can be directed to the corresponding author/s.

## Ethics Statement

The studies involving human participants were reviewed and approved by the Ethical Committee of the University Hospital Campus Bio-Medico of Rome (28.16TS Com Et CBM). Informed consent was not required for the retrospective design of the study. The ethics committee waived the requirement of written informed consent for participation.

## Author Contributions

SS, SA, JD, and CB wrote the manuscript and conceived and designed the study. SA, MC, and EV analyzed the data. EV, MF, LL, GA, GB, and SC collected and provided the sample for this study. JD, AA, JA, CB, and RM revised, optimized, and fixed the manuscript. All authors have read and approved the final submitted manuscript.

## Conflict of Interest

The authors declare that the research was conducted in the absence of any commercial or financial relationships that could be construed as a potential conflict of interest.

## Publisher’s Note

All claims expressed in this article are solely those of the authors and do not necessarily represent those of their affiliated organizations, or those of the publisher, the editors and the reviewers. Any product that may be evaluated in this article, or claim that may be made by its manufacturer, is not guaranteed or endorsed by the publisher.

## Footnotes

1
https://www.Rproject.org/2019
